# Supplementary material for: Direct supercritical angle localization microscopy for nanometer 3D superresolution
Source: Nat Commun. 2021 Feb 19;12:1180. doi: 10.1038/s41467-021-21333-x (PMC7896076; doi:10.1038/s41467-021-21333-x)
Supplement: Supplementary file 2 — Supplementary Information [file 41467_2021_21333_MOESM2_ESM.pdf]

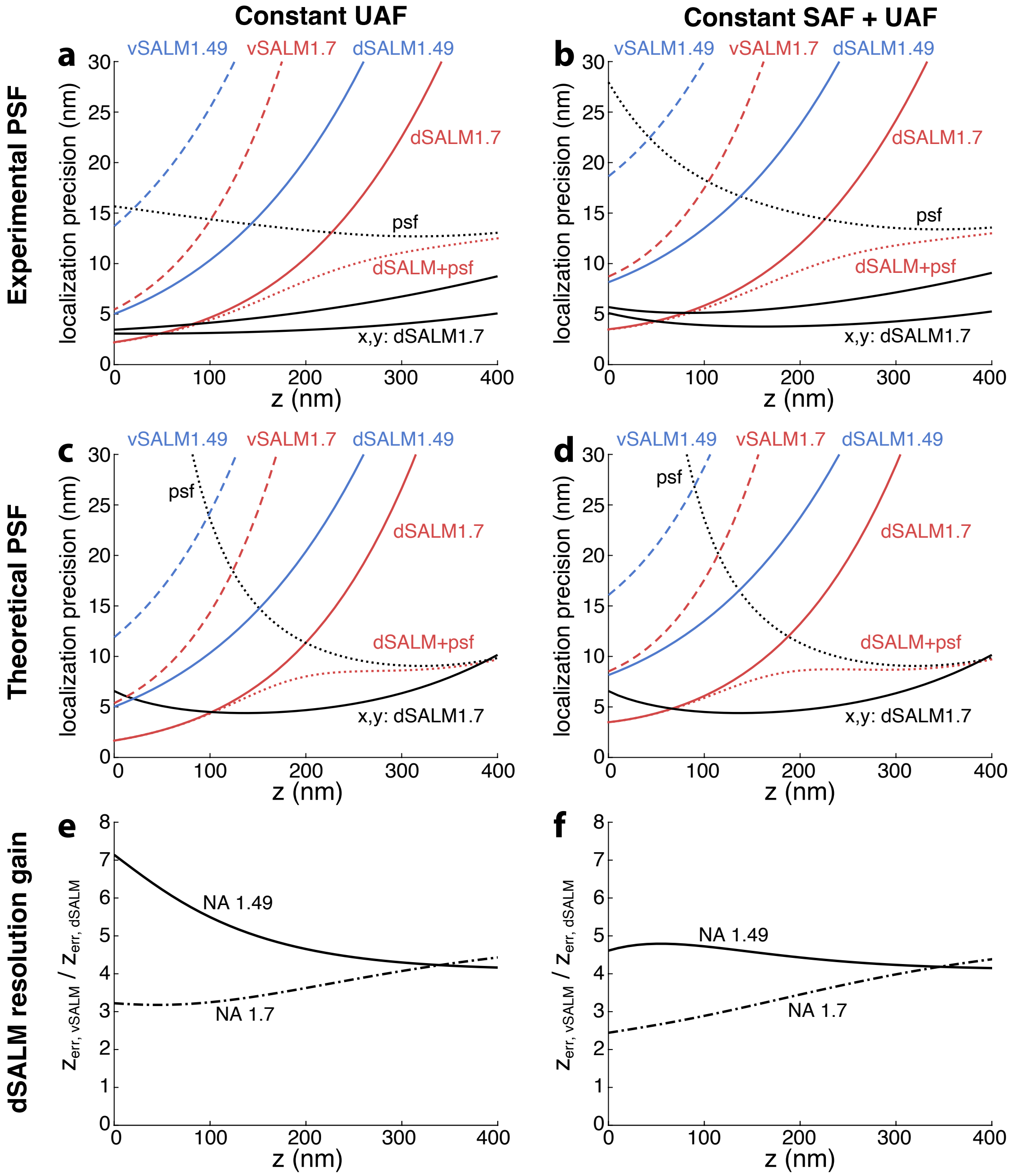

Supplementary Figure 1: Theoretical resolution of supercritical-angle localization microscopy for vSALM (dashed lines, a-d) and dSALM (solid lines, a-d) with an NA 1.49 (blue) and an NA 1.7 (red) objective. All values are axial localization precisions, unless noted otherwise. Point-spread-function (psf) denotes the z localization precision obtained from fitting the PSF shape as common for astigmatic SMLM. Comparison of experimentally derived PSF with astigmatism (a,b) and theoretical PSF without astigmatism calculated according to Zelger et al., Biomed. Opt. Express 11 (2020) (c,d), except for dSALM1.49 in a,b that is based on the theoretical PSF of c,d. (e,f) ratio of z localization precisions between vSALM and dSALM, which corresponds to the relative gain in resolution of dSALM vs. vSALM. Calculations are based on the theoretical PSF. (a,c,e) assume constant UAF, as an approximation for fluorophores with a low quantum yield where SAF competes mostly with non-radiative decay. (b,d,f) assume constant total fluorescence, as an approximation for fluorophores with a high quantum yield.

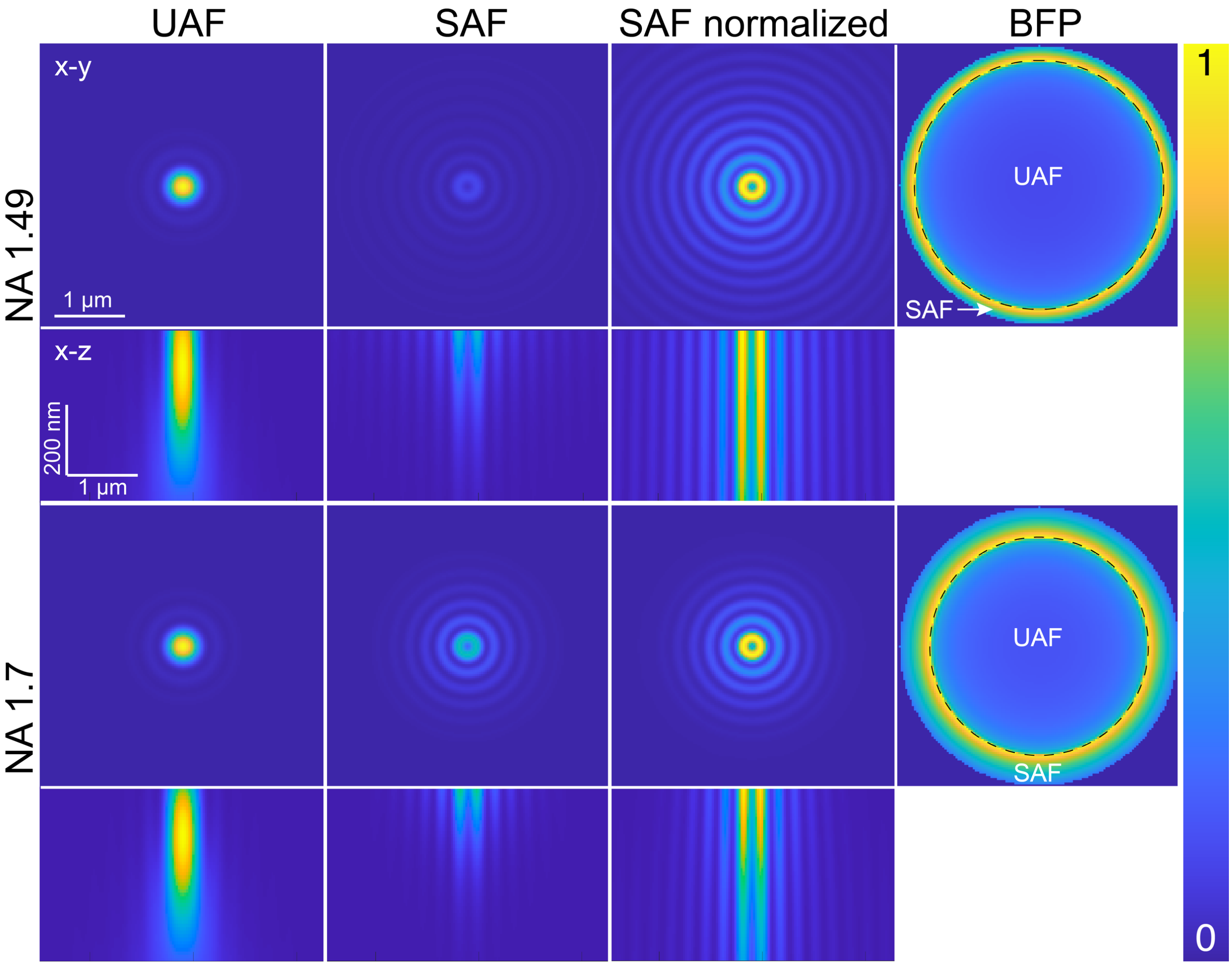

Supplementary Figure 2: Calculated PSFs for the NA 1.49 and the NA 1.7 objective in the dSALM configuration for the undercritical angle fluorescence (UAF) and supercritical angle fluorescence (SAF) channel, and the corresponding back focal plane intensity distribution. The emitter is at the coverslip (z=0). Calculations based on code from Zelger et al., Biomed. Opt. Express 11 (2020). The SAF PSF displays strong diffraction rings leading to a large PSF size, especially visible in the 'SAF normalized', where the intensity is normalized to its maximum. For 'SAF normalized' x-z, each z position is normalized to the summed intensity of the corresponding x-y plane, highlighting the shape of the PSF, but neglecting the decay of the SAF field. In the calculated BFP images, the mirror separating UAF and SAF is denoted with a dashed line.

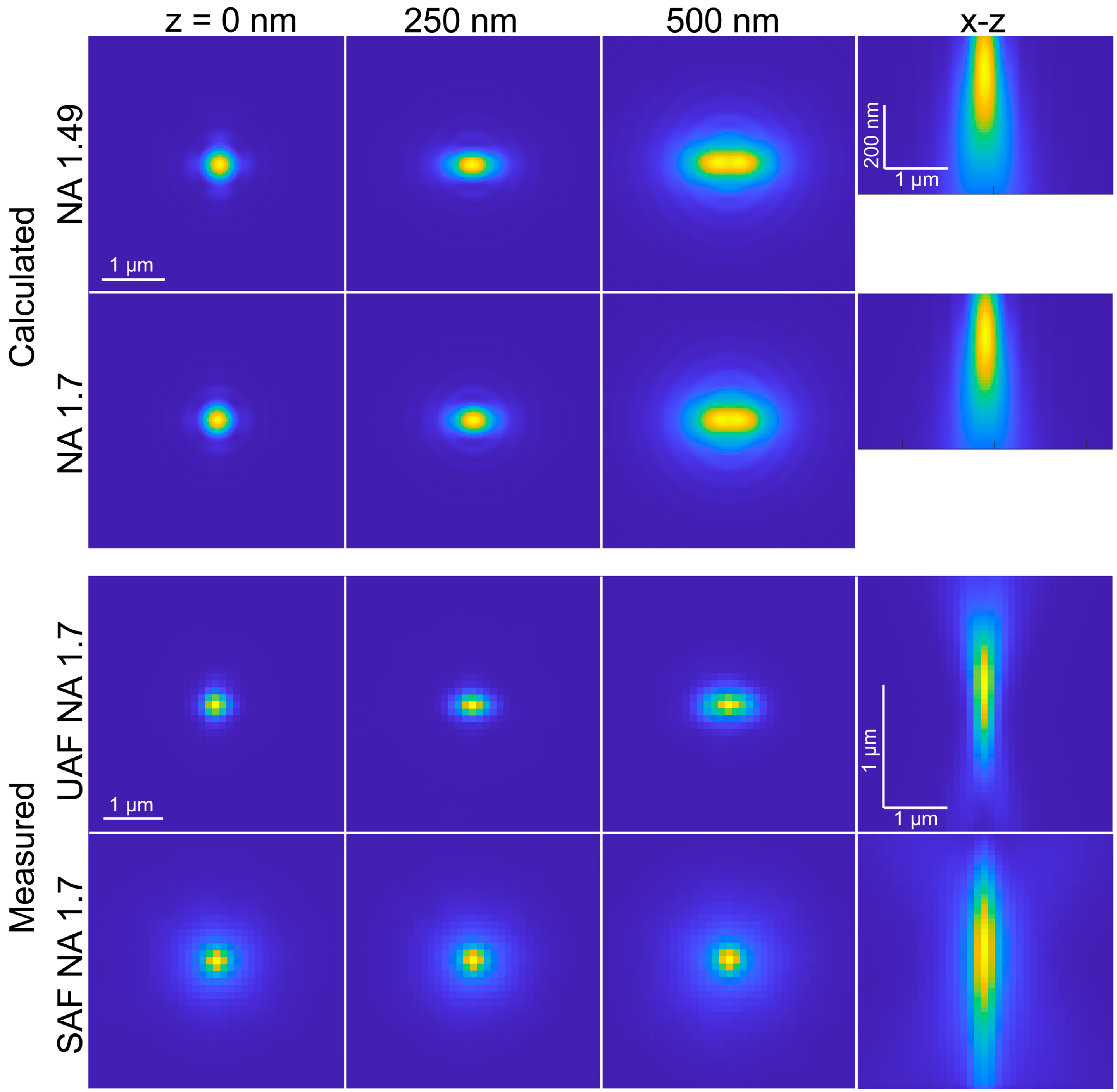

Supplementary Figure 3: Astigmatic PSF and experimental PSF. Calculated PSFs for the NA 1.49 and the NA 1.7 objective for the undercritical angle fluorescence (UAF) channel including weak astigmatism for different z positions of the point-emitters. Calculations based on code from Zelger et al., Biomed. Opt. Express 11 (2020). Each calculated PSF slice is normalized by its maximum amplitude.
Measured PSFs for the NA 1.7 objective in the UAF and supercritical angle fluorescence (SAF) channel. Experimentally derived point spread function (PSF) calculated from z-stacks of 100 nm beads, average of 40 beads. Each measured PSF slice is normalized to its brightest pixel.

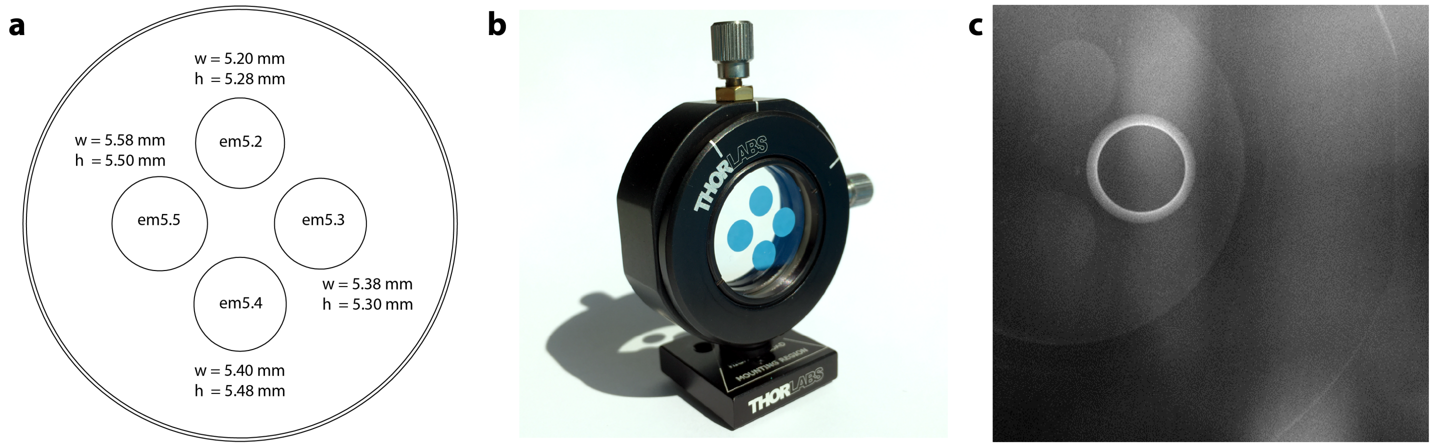

Supplementary Figure 4: Elliptical masks. a, Specification of the masks dimension, designed to be placed at an angle of 10° in the beam path (w: width, h: height, em: elliptical mask.). **b,** Picture of the four elliptical masks deposited on the glass window and housed in a 2D translation mount. **c,** Camera image of the BFP with stray light highlighting the masks and the holder.

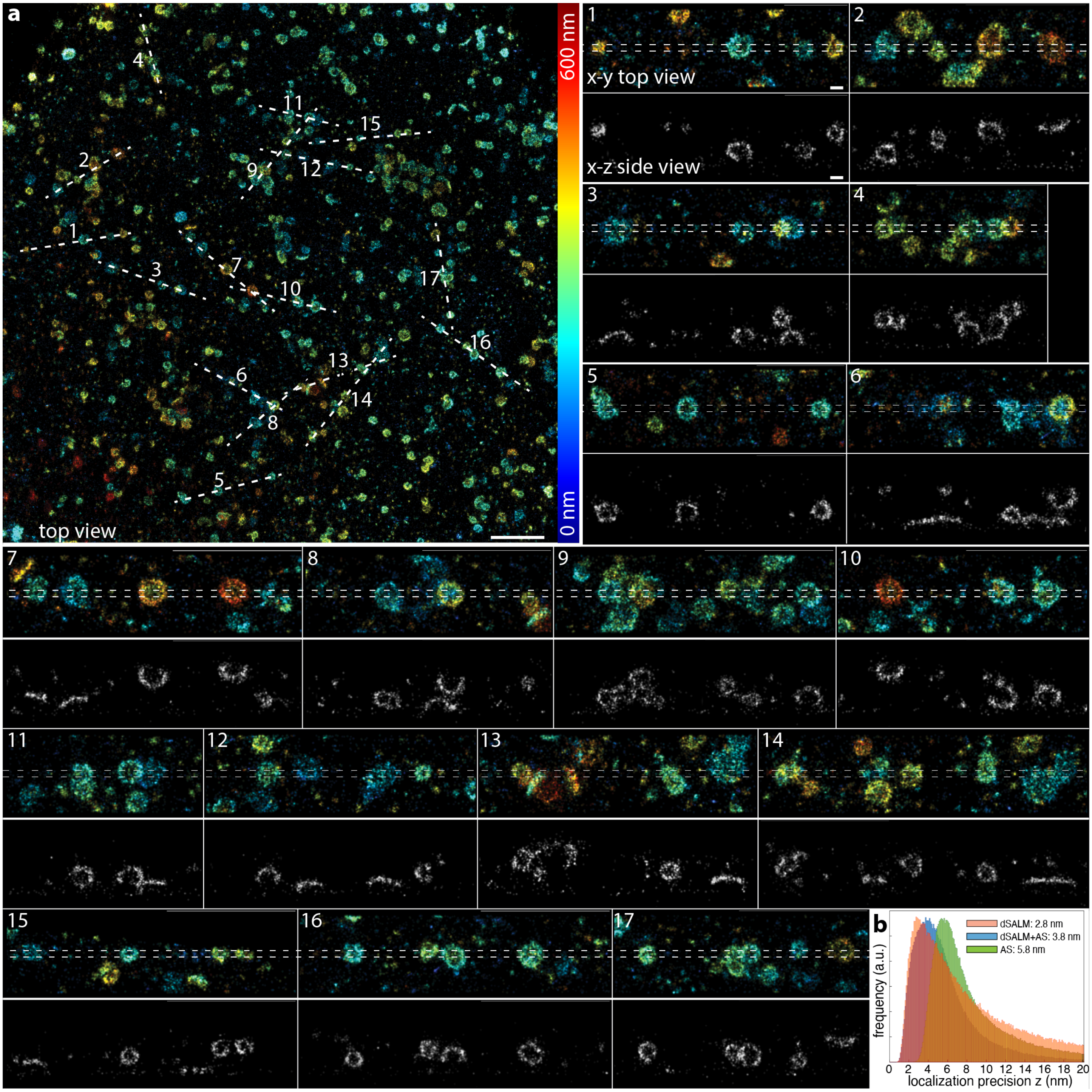

Supplementary Figure 5: Clathrin coated pits. a, Clathrin coated pits, labeled with anti clathrin heavy and light chain primary and fast DNA-PAINT secondary antibodies. Localizations are color-coded according to their z positions. Insets represent the various sites of the overview image in x-y top view (top) and x-z side view (bottom). **b,** Histograms of the localization precision (CRLB) of the z-coordinate z_{dSALM} for dSALM (orange), z_{av} for combined dSALM and astigmatism (blue) and z_{as} for astigmatism alone (green). Scale bars 1 µm (a), 100 nm (1).

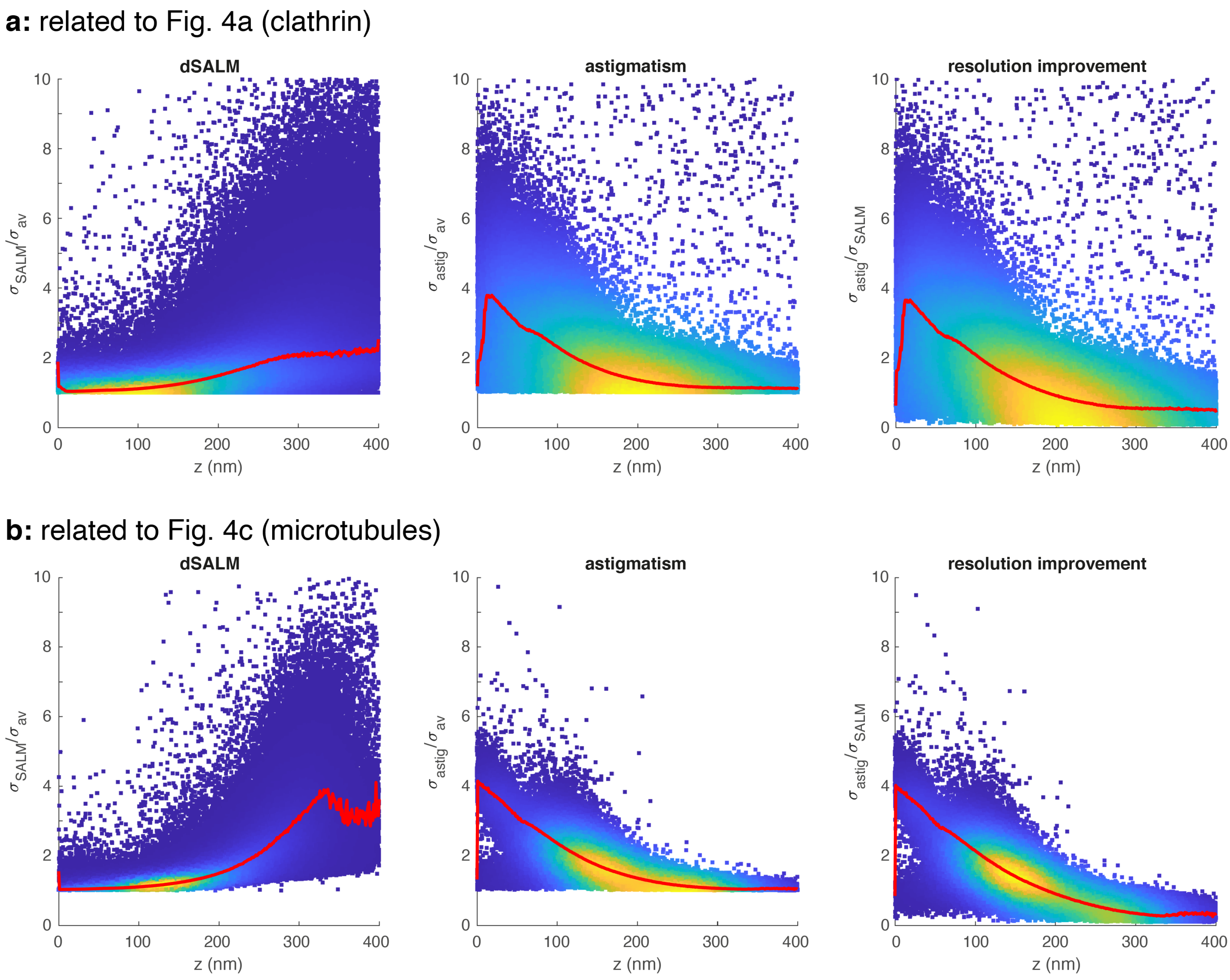

Supplementary Figure 6: Axial localization precision for clathrin (**a**, Fig. 4a, Supplementary Figure 5), and microtubules (**b,** Fig. 4c). As every localization has random photon number, we normalized the localization precisions by the average localization precision σ_{av}. Left: dSALM localization precision σ_{SALM}, center: astigmatism localization precision σ_{astig}. For small z (<200 nm) σ_{SALM} is close to σ_{av} and thus determines the overall localization precision σ_{av}. For larger z, σ_{astig} is close to σ_{av} and determines the overall localization precision. The right panel shows the ratio of $\sigma_{astig}/\sigma_{SALM}$ and thus the resolution improvement of dSALM alone vs astigmatism alone. Red lines denote a running median.

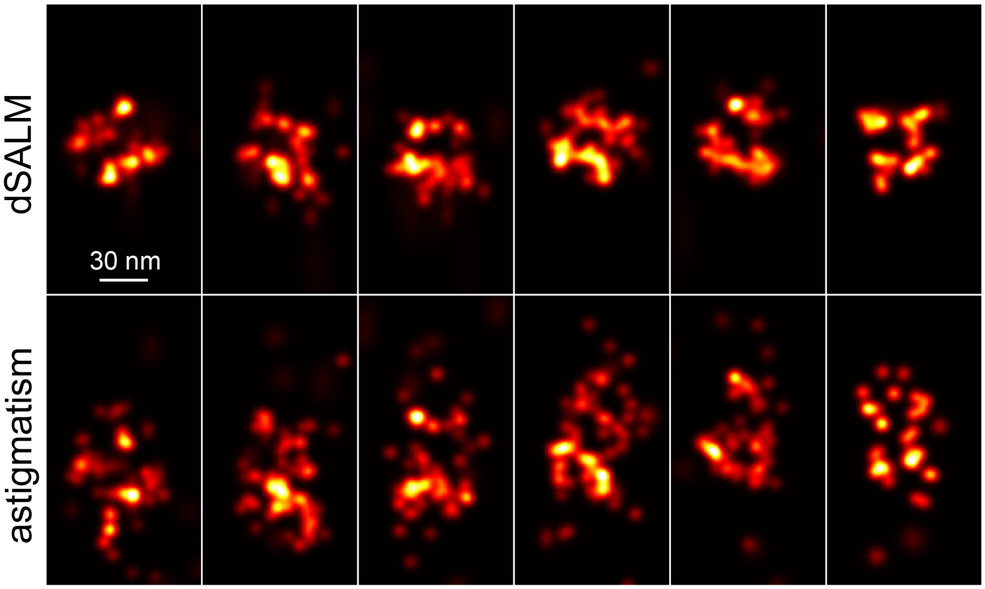

Supplementary Figure 7: Comparison of dSALM with astigmatism on 30 nm DNA origami structures (compare Fig. 3b). Side-view reconstructions, rotated around the z-axis to result in side views on the sometimes tilted structures. Same view for dSALM reconstruction and astigmatism reconstruction (undercritical angle fluorescence (UAF) channel only). As we use rather weak astigmatism to keep a high lateral resolution, optimized microscopes with stronger astigmatism could result in a higher resolution. Representative images from 2 independent replicates are shown.

SALM Analysis pipeline using SMAP

Installation of SMAP

SMAP can be downloaded at https://github.com/jries/SMAP. A compiled version for PC and Mac, as well as documentation and installation instructions are available at https://rieslab.de. For installation and general usage please follow the SMAP User guide:
https://www.embl.de/download/ries/Documentation/SMAP_UserGuide.pdf
If you are not familiar with SMAP, we recommend to get started with:
https://www.embl.de/download/ries/Documentation/Getting_Started.pdf

SALM Pipeline

Calibrate experimental PSF from bead stacks

1. Acquire z-stacks of 10 – 20 different FOVs of 100 nm beads with the following settings:
    a. z-range: -1.2 µm – 1.2 µm
    b. ∆z = 30 nm (also values as 20 nm or 50 nm work)
    c. single-mode excitation
    d. acquisition in conventional gain (to increase the signal to noise ratio)
2. Start SMAP.
3. Find the plugin *'calibrate3DsplinePSF'* either from the [menu] → [Plugins] → [Analyze] → [sr3D] or in the [Analyze] tab, [sr3D] sub-tab and press *'Run'*. Now the GUI for the spline model calibration opens.
4. *'Select camera files'* to load all the bead stacks. A new window opens. Here press *'add dir'* and select all directories in the bead directory. Press *'Done'*.
5. Set the parameters according to Figure 1.
    a. As we have dual-channel (UAF and SAF) data in two parts of the camera we choose the 3D modality: *'global 2 channel'*.
    b. For nicer visualization of the validation of the model you can select *'bi dir'* to activate bi-directional fitting.
    c. Set the *'distance between frames'* in the stack (in nanometers).
    d. To not select overlapping PSFs, *'minimum distance'* of 17 pixels is chosen
    e. Select main channel as left *'u/l'* which is the UAF channel on the left of the camera image for our setup. Also specify that the two channels are *'right-left'* oriented.
    f. Let SMAP determine the EM gain and mirror from the metadata. Otherwise, set here if beads were acquired with the conventional or check for *'EM gain and mirror from metadata'*.
6. Press *'Calculate bead calibration'*. It roughly takes around 5 minutes for the process to generate the PSF model.
    This analysis is automatically saved in output file directory with example_3dcal.mat and

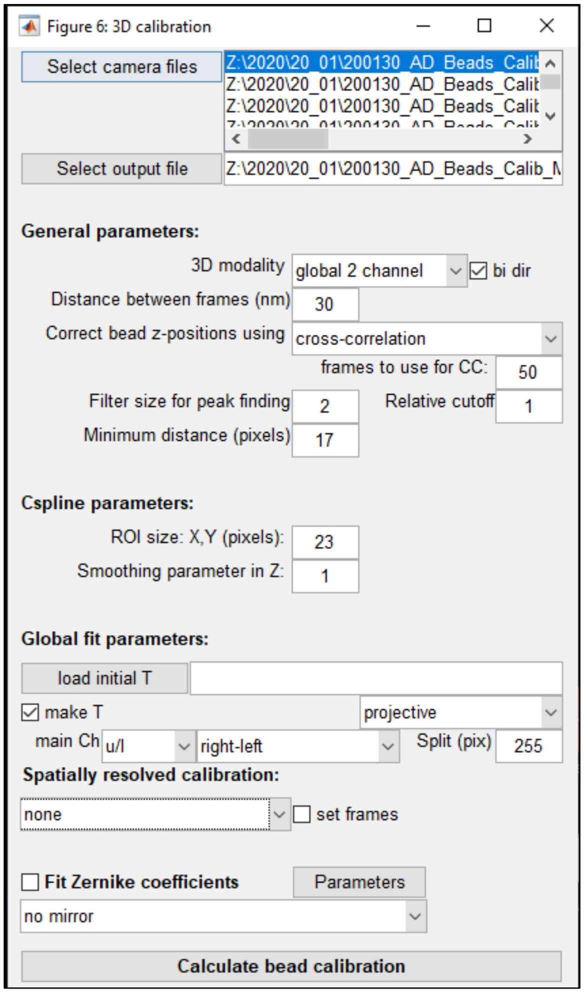

Figure 1: 'calibrate3DsplinePSF' GUI

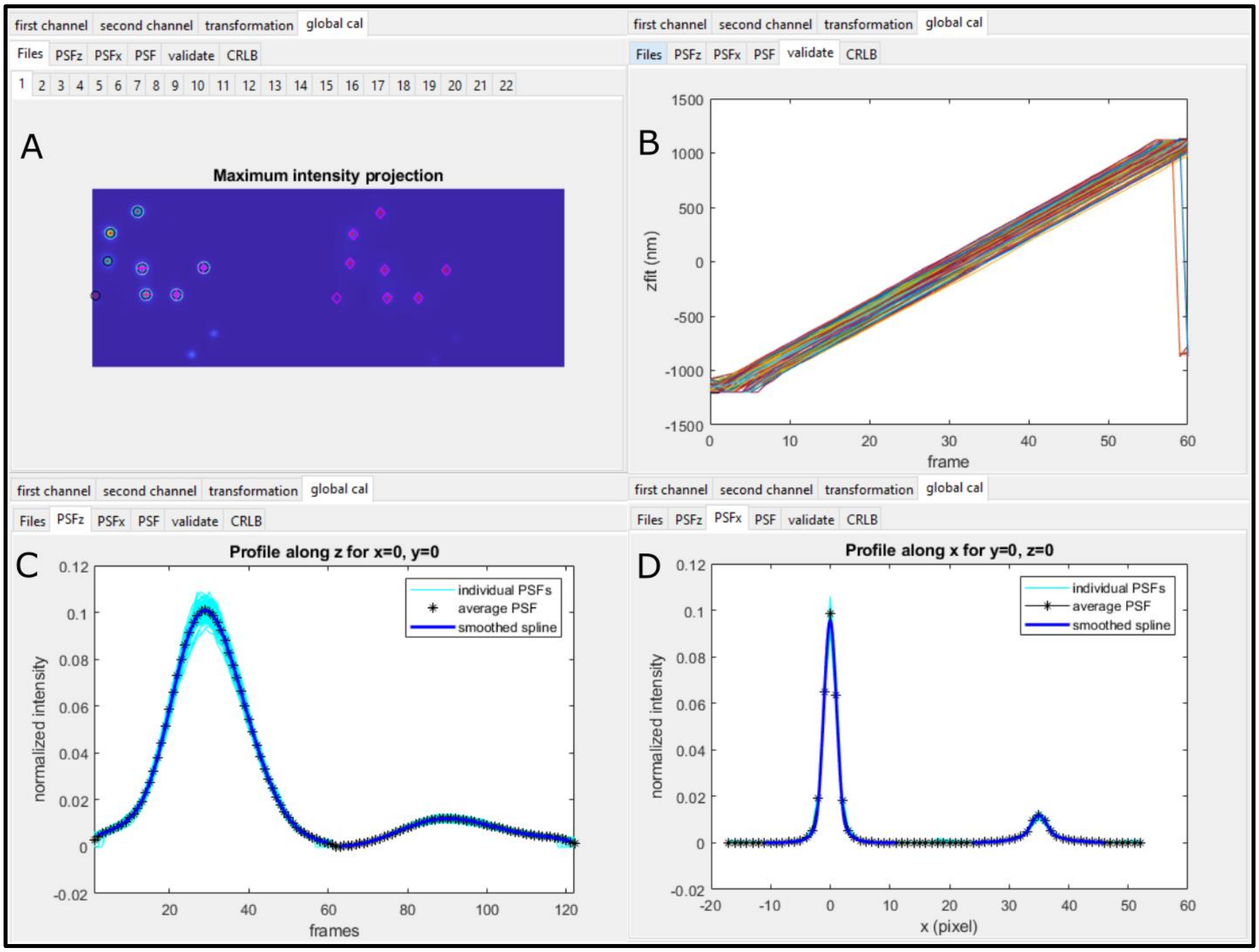

Figure 2: Output of the calibration file. A) Shows the selected beads in two channels from stack files for calibration. B) Shows the fitting of the individual beads by the model. C) Shows the cross-sectional profile along z-axis of the model representing individual beads. D) Shows the cross-sectional profile along x-axis of the model representing individual beads.

example_3dcal.fig. Reopening the example_3dcal.fig, one can compare the tabs of the output figure with those in Figure 2.

Fit 3D SALM data (both UAF and SAF channels)

1. Check in the SMAP GUI at the bottom of the [Localize] → [Fitter] that the fitting workflow *'fit_fastsimple'* is selected. Otherwise load that workflow by pressing *'Change'*.
2. Select [Localize] → [Input Image] → click on *'Load image'* to load the files from the directory. Check *'Online analysis'* if you want to fit during the acquisition.
3. Set the parameters for the initial estimation of single molecule positions in the [Peak Finder] tab according to Figure 3C.
   a. If data is acquired using EM gain mode then check *'EM mirror'*.
   b. Use ToolTips (hover mouse over control) to get information about specific parameters.
   c. Convolution using Difference of Gaussian *'DoG'* is set to 1.2. Typically, *'dynamic(factor)'* is set to 1.5.
   d. By pressing *'Preview'* after selecting a frame with the slider next to it an image will open which will show the positions of the found localizations. Use this preview to optimize peak finding parameters.

   e. Select *‘ROI to exclude*’ to draw a rectangular area on the region between SAF and UAF. ‘Double click’ on the ROI drawn on the image to restrict fitting of single molecules in the boundary between two channels (Figure 3C).
4. Select [Localize] → [Fitter]. Set parameters according to Figure 3B.
   a. select *‘Spline’* and click on *‘Load 3D cal’* load the bead calibration file that you just generated.
   b. The size of the ROI in which the fitting of single molecules is performed is set to be 13 pixels.
   c. Check ‘RI mismatch’ and set to 0.6 to rescale the fitted z-positions.
   d. Select a large frame (e.g. 5000 as the first frames are still too dense to optimize fitting parameters) and press *‘preview’*. The results should be comparable to those in Figure 3C.
5. Select [Localize] → [Localizations]. Ensure all the parameters under *‘Filter ([min max])’* are unchecked.
6. Press *‘Localize’* and wait for the fitting to be done (this might take up to one hour depending on your computer).
7. You a have a first look at the results by pressing *‘Render’* in the [Render] tab.
8. Separately, one can directly load an existing fitted file with ‘.sml’ extension. Go to [File] →’Load’ and then proceed with the rendering section for further analysis.

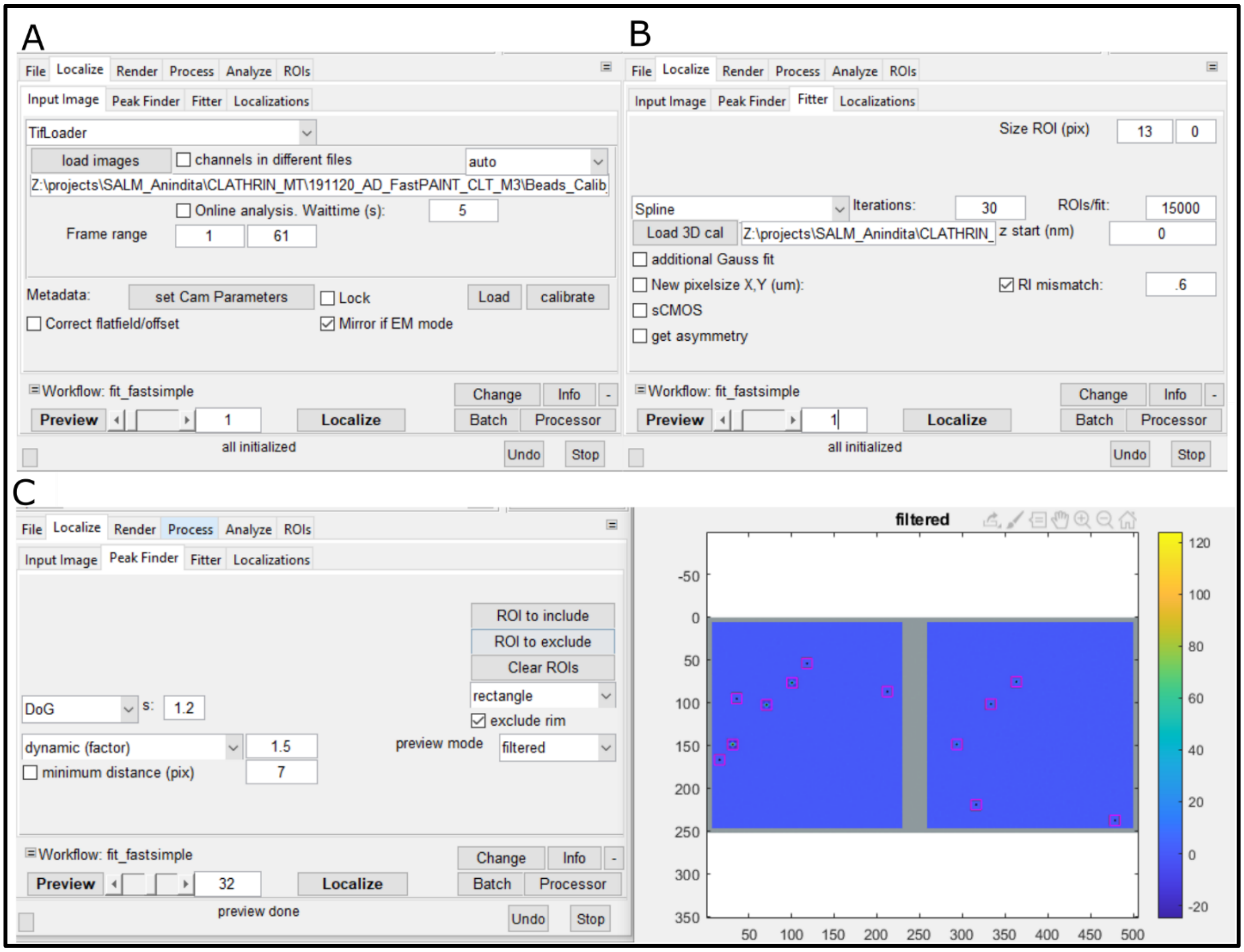

Figure 3: A) Shows the parameters to be chosen while loading the input file. B) Shows the parameters for the Fitter tab. C) Shows the adjusted parameters for the Peak Finder tab.

Rendering of localizations

1. Check in the SMAP GUI at the bottom of the [Render]. In order to remove incorrect localizations, rendering is done based on specific parameters according to Figure 4.
2. Go to [Render] → *'Layer1'* and click on '+' next to add a new layer namely *'Layer2'*. Two layers are created, with Layer1 being filtered strictly as compared to Layer2. This is done to later assign Layer1 to the UAF channel and Layer2 to the SAF channel during creation of the transformation file. The SAF channel consists of less signal and is thus is prone to removal of localizations by strict filtration.
3. On top right corner of SMAP GUI, you can select which layer you want to display. Checking both layers will superimpose one on the other.
4. Ensure Layer1 is selected for display and also in the layer selection panel. Render based on the parameters similar to Figure 4.
    a. Select the likelihood-filter '*LLrel*' (Figure 4a). Activate the '*LLrel*' filter by checking filter. Set the filter to exclude the accumulation of localizations (shown as a green bar) on the left side of the histogram.
    b. If an overview of the image is shown instead of the table, click *'OV->filter'*
    c. Set 'locprec' around 10-15nm
    Note: These filtering parameters vary with every dataset. Adjust the parameters carefully to avoid over-filtering of localizations.
5. Also filter (if needed) on Layer2 by 'likelihood' only. Strong filtering in this layer will lead to loss in significant localizations.
6. Ensure the size of image for both the layers are the same. Then check on 'Layer1' and 'Layer2' on top right of SMAP GUI to overlay one on the other.

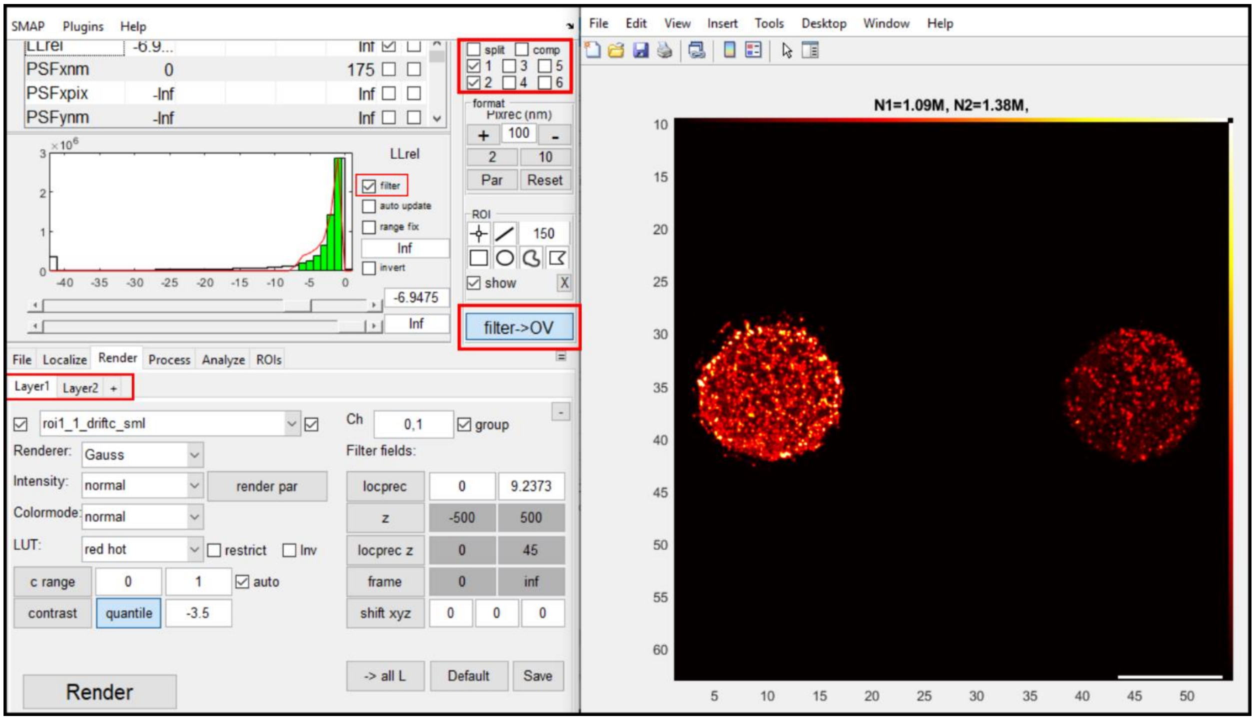

Figure 4: Shows the GUI to render the fitted dataset.

Calculate transformation between UAF and SAF channels and extract photon counts from camera image

1. This step assigns the intensities in both undercritical and supercritical channel. Select [menu] → [plugins] → [Process] → [Assign2C] → [IntensitiesforSALM]. This open a new window *'GetIntensitiesSALM'.*
2. Select the parameters as shown in Figure 5A.
3. UAF channel is assigned to layer1 and SAF channel to layer2. The raw image stack is uploaded along with the 3D calibration file measured before.
4. *'roisize'* is set to 17 pixels for correlation and '*BG free fit'* is selected. Check *'normalize image to Poisson noise'* and *'Show results'*.
5. It takes some time to create the correlation between two channels and generate the transformation file. It is automatically saved in the working directory with the extension *'_dc_sml'*.

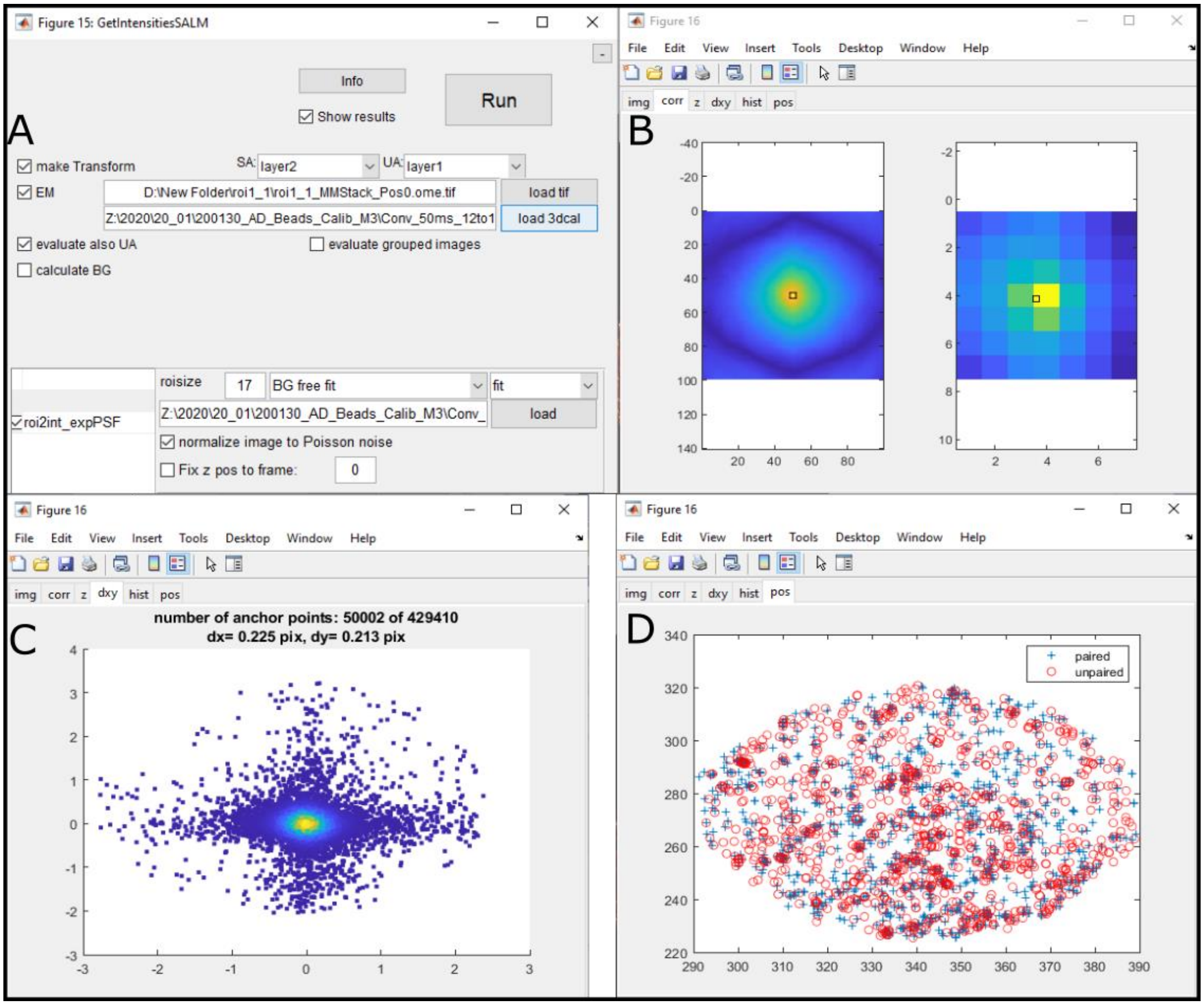

Figure 5: A) Shows the GUI 'GetIntensitiesSALM' to calculate transformation. B) Shows that the correlation should have a clear peak. C) Shows that the errors should form a clear maximum in the center, the standard deviations dx and dy should be << 1 pixel. Shows that the paired localizations should span the whole field of view.

Perform drift correction

1. On the SMAP GUI select [Process] → [Drift] → *'drift correction X,Y,Z'.*
2. Ensure on top right corner of SMAP GUI, only Layer1 is checked for display. Only the UAF part is taken for drift correction as shown in Figure 6.
3. Keep the parameters for drift correction as shown in Figure 6. The following parameters are important:
    a. *'timepoints'* for xy-drift: The number of timepoints the superresolved image is split into for cross-correlation.
    b. *'pixrec nm'* for xy-drift: Pixelsize for the reconstructed images
    c. Tick the box for *'Correct z-drift'* if you are working with 3D data.
    d. *'Timepoints z'* and *'z binwidth nm'* are analogous to above mentioned values
    e. Tick the box in front of *'Save driftcorrected SML'* to automatically save a new file after drift correction.
4. Click on *'Run'* and evaluate the quality of the drift-correction in the window with figures that automatically opened.
5. The drift-corrected localizations are by default automatically saved as a '_driftc_sml.mat' file.

NOTE: Processed file are automatically saved until drift correction. Loading '_driftc_sml.mat' file means the following steps of CRLB calculation and zSALM have to be executed. However, after final zSALM step one can always save with the '*SMLMsaver*' in the [File] tab with appropriate filename.

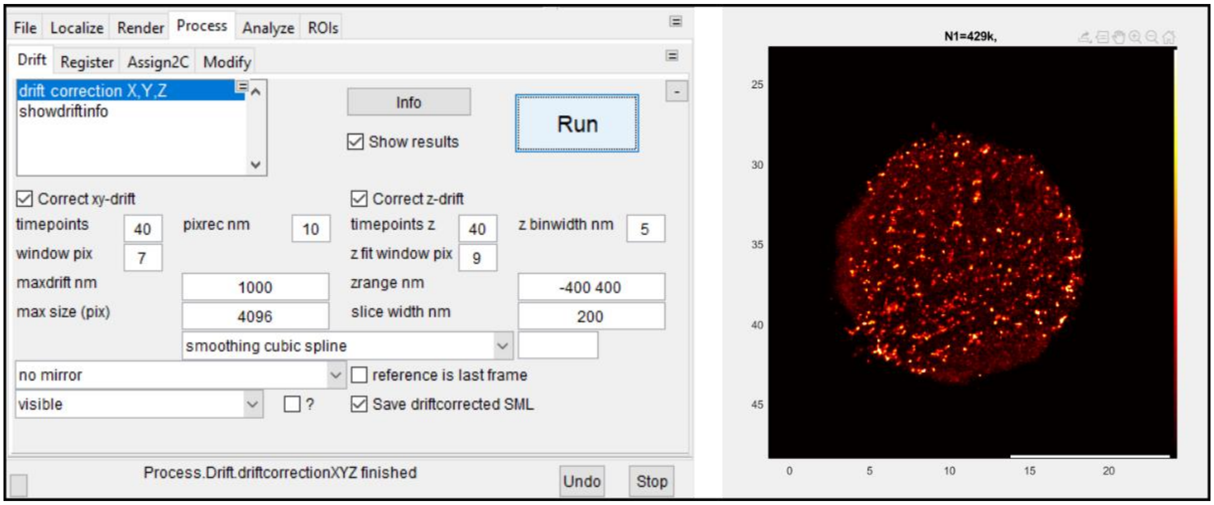

Figure 6: GUI for drift correction.

Calculate the CRLB for the intensities

1. On the SMAP GUI open the plugin [Analyze] → [sr3D] →SALM_getCRLB as shown in Figure 7.
2. Load the _3dcal.mat bead calibration file.
3. Make sure that the correct fields are selected for SAF and UAF intensities and background.
4. You can also change the refractive index rescaling here.
5. Click *'Run'*. This plugin might take minutes to hours depending on the number of localizations to calculate the CRLB.

Calculate the z-coordinates from the intensities

1. Open the plugin [Analyze] → [sr3D] →zSALM.
2. Keep the parameters as shown in Figure 8. Make sure that the correct fields are selected for SAF and UAF intensities and background. If needed, adjust the parameters.
3. The following parameters are assigned to calculate z position:
    a. *'Intensity SA'* intensities from supercritical region: psf_ns
    b. *'bg SA'* background from supercritical region: psf_bgs
    c. *'Intensity UA'* intensities from undercritical region: psf_nu
    d. *'bg UA'* background from undercritical region: psf_bgu
    e. *'n buffer'* for refractive index of the buffer: 1.33
    f. *'n immersion'* for refractive index of the immersion oil: 1.78
    g. *'lambda'* for emission wavelength: 680 nm
    h. *'NA objective'*: 1.70
    i. *'NA mask'*: 1.35 (our mirror was slightly larger than the NA of 1.33 that would perfectly separate UAF and SAF).
    j. *'z range'* over distance from coverslip: -400 nm(below) to 800nm (above)
    k. *'SA/UA ratio on coverslip'*: 1
    l. *'Field znm'*: weighted average (Z as weighted average of Z_{SALM} and Z_{AS})
4. Click *'Run'*.
5. Save the processed superresolution data with the '*SMLMsaver*' in the [File] tab.

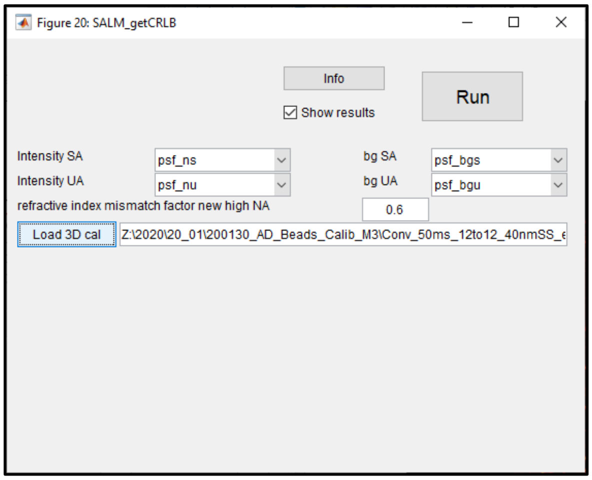

Figure 7: GUI to calculate Cramér–Rao lower bound (CRLB).

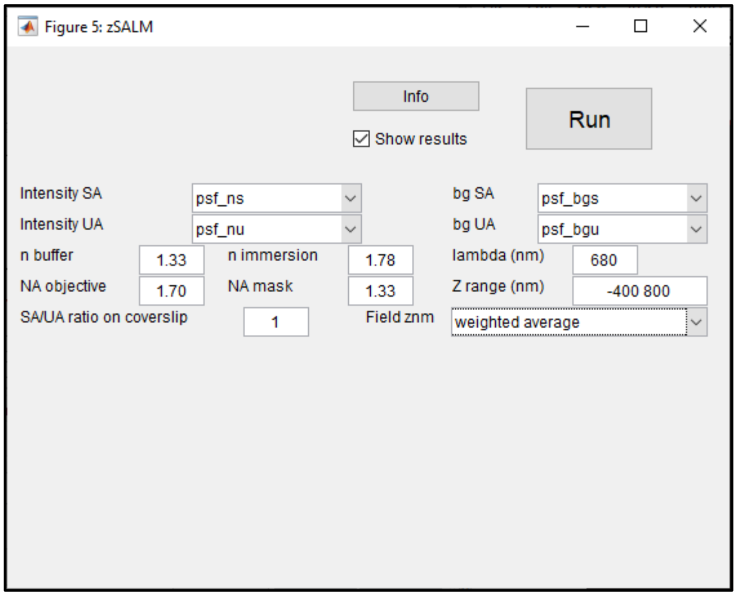

Figure 8: GUI 'zSALM' to calculate the z-coordinate for every single molecule blinking event.

Rendering of 3D superresolution data

Before analysis, render the 2D images with color coding according to zSALM. Ensure the image is correctly filtered to exclude incorrect localizations. Filtering can be achieved by setting boundaries for the parameters like localization precision (typically 0-10 nm in UAF channel), frames and log-likelihood.

1. Go to [Render] tab and ensure *'Layer1'* is selected. The UAF part is rendered and analyzed.
2. '*Colormode*' is set to 'z' indicating that the localizations are color coded based on their z positions.
3. The '*LUT*' (look up table) is set to 'jet'
4. Adjust the '*c range*' to assign values that directly corresponds to the LUT.
5. Click on '*Render*' to implement your assignments.
6. An overview of this step is shown in Figure 9.

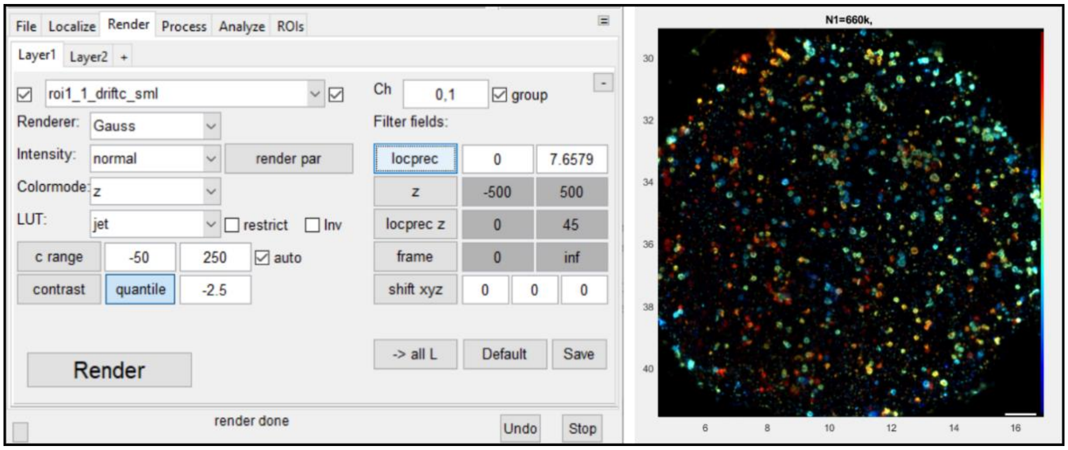

Figure 9: Render the 2D images with color coding as per the z position of the localizations.

Analysis of 3D superresolution data

1. Zoom into a region of interest on the image with nice looking structures. For this example dataset we zoom into nice looking clathrin mediated endocytotic pits.
2. Open the plugin [Analyze] → [sr3D] →[Viewer 3D].
3. Draw a line ROI over the nice looking structures whose cross-sectional view can be visualized. The width of the line ROI can be modified as shown in Figure 10. The render parameters (color, filtering,..) are the same as for the main rendered image (as set in the [Render] '*Layer1*' tab).
4. Set *'z,zerr'* values to '*znm_SALM*' and '*locprecznm*'.
5. Set 'zmin/zmax' so that the structures are in the center of the z range. For this example dataset we set it from -100 nm to 400 nm.
6. You can drag the line ROI and the side view reconstruction will automatically follow. You can click on [X] to delete current ROI and select a new one as shown in Figure 10.
7. You can start a dynamic rotation by pressing *'Animate'*. To reset to the side view press '*Polar angle theta*'. You can change the direction of translation by selecting '*horizontal/vertical'*.
8. Press on '*save movie*' to save the rotating animation.
9. The animation is saved as a tiff stack. You can use Fiji to export it to avi or mp4.
10. For more information on the 3D viewer see the SMAP User guide.

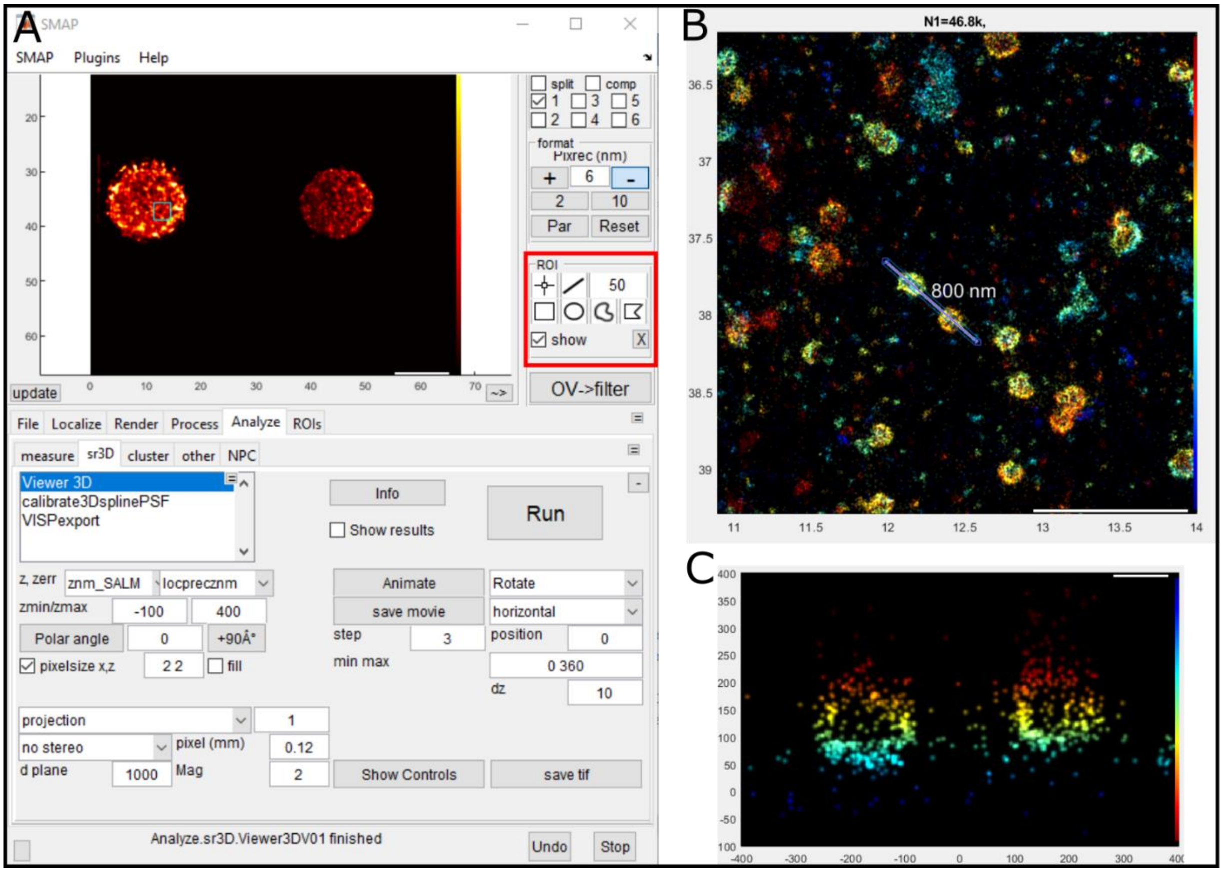

Figure 10: A) GUI for 3D rendering. Highlighted region shows the different ROI formatting options. B) A line ROI on the main rendered image C) Side-view rendering of the localizations in the line ROI.
