# Supplementary material for: Direct supercritical angle localization microscopy for nanometer 3D superresolution
Source: Nat Commun. 2021 Feb 19;12:1180. doi: 10.1038/s41467-021-21333-x (PMC7896076; doi:10.1038/s41467-021-21333-x)
Supplement: Supplementary file 3 — Reporting Summary [file 41467_2021_21333_MOESM3_ESM.pdf]

nature research

Corresponding author(s): Jonas Ries

Last updated by author(s): Jan 10, 2021

Reporting Summary

Nature Research wishes to improve the reproducibility of the work that we publish. This form provides structure for consistency and transparency in reporting. For further information on Nature Research policies, see our Editorial Policies and the Editorial Policy Checklist.

Statistics

For all statistical analyses, confirm that the following items are present in the figure legend, table legend, main text, or Methods section.

n/a	Confirmed	
☒	☐	The exact sample size (*n*) for each experimental group/condition, given as a discrete number and unit of measurement
☒	☐	A statement on whether measurements were taken from distinct samples or whether the same sample was measured repeatedly
☒	☐	The statistical test(s) used AND whether they are one- or two-sided<br>*Only common tests should be described solely by name; describe more complex techniques in the Methods section.*
☐	☒	A description of all covariates tested
☒	☐	A description of any assumptions or corrections, such as tests of normality and adjustment for multiple comparisons
☒	☐	A full description of the statistical parameters including central tendency (e.g. means) or other basic estimates (e.g. regression coefficient) AND variation (e.g. standard deviation) or associated estimates of uncertainty (e.g. confidence intervals)
☒	☐	For null hypothesis testing, the test statistic (e.g. *F*, *t*, *r*) with confidence intervals, effect sizes, degrees of freedom and *P* value noted<br>*Give P values as exact values whenever suitable.*
☒	☐	For Bayesian analysis, information on the choice of priors and Markov chain Monte Carlo settings
☒	☐	For hierarchical and complex designs, identification of the appropriate level for tests and full reporting of outcomes
☒	☐	Estimates of effect sizes (e.g. Cohen's *d*, Pearson's *r*), indicating how they were calculated

Our web collection on statistics for biologists contains articles on many of the points above.

Software and code

Policy information about availability of computer code

Data collection	Micro-Manager 2.0
Data analysis	MATLAB 2020a, SMAP V200623 (custom data analysis software) from github.com/jries/SMAP.

For manuscripts utilizing custom algorithms or software that are central to the research but not yet described in published literature, software must be made available to editors and reviewers. We strongly encourage code deposition in a community repository (e.g. GitHub). See the Nature Research guidelines for submitting code & software for further information.

Data

Policy information about availability of data

All manuscripts must include a data availability statement. This statement should provide the following information, where applicable:

- Accession codes, unique identifiers, or web links for publicly available datasets
- A list of figures that have associated raw data
- A description of any restrictions on data availability

Data are available from the corresponding author (J.R.) upon request. An example data set including detailed instructions for analysis can be downloaded at https://www.embl.de/download/ries/dSALM/. Source data are provided with the manuscript.

Field-specific reporting

Life sciences study design

All studies must disclose on these points even when the disclosure is negative.

Sample size	30 nm nano-pillars: 5 datasets from three independent experiments. 80 nm nano-pillars: 9 datasets from three independent experiments. 50 nm beads: 98 datasets from two independent experiments. The size of these data sets allowed for a meaningful statistical analysis. Clathrin: 3 datasets from 3 independent experiments. Microtubules: 2 datasets from 2 independent experiments. These data demonstrated consistency and reproducibility of our technique.
Data exclusions	No data excluded.
Replication	Samples for individual experiments were independently prepared and results compared to proof reproducibility. All replication attempts were succesful.
Randomization	n/a Our experiments were primarily concerned with technique development and optimization of measurements, rather than comparison of groups. Therefore blinding and randomization is not applicable to our work.
Blinding	n/a Our experiments were primarily concerned with technique development and optimization of measurements, rather than comparison of groups. Therefore blinding and randomization is not applicable to our work.

Reporting for specific materials, systems and methods

We require information from authors about some types of materials, experimental systems and methods used in many studies. Here, indicate whether each material, system or method listed is relevant to your study. If you are not sure if a list item applies to your research, read the appropriate section before selecting a response.

Materials & experimental systems

n/a	Involved in the study	
☐	☒	Antibodies
☐	☒	Eukaryotic cell lines
☒	☐	Palaeontology and archaeology
☒	☐	Animals and other organisms
☒	☐	Human research participants
☒	☐	Clinical data
☒	☐	Dual use research of concern

Methods

n/a	Involved in the study	
☒	☐	ChIP-seq
☒	☐	Flow cytometry
☒	☐	MRI-based neuroimaging

Antibodies

Antibodies used	rabbit anti-clathrin heavy chain antibody, ab21679, Abcam; rabbit anti-clathrin light chain antibody, sc28276, Santa Cruz Biotechnology; mouse-anti-beta-tubulin antibody, T5293, Sigma-Aldrich; donkey anti-mouse secondary secondary antibody, 715-005-151, Jackson ImmunoResearch; donkey-anti-rabbit secondary antibody,711-005-152, Jackson ImmunoResearch
Validation	All antibodies resulted in strong and specific staining showing the expected structures in microscopy images.Validation according to manufacturer: ab21679 validated for ICC on human cells. sc28276 not validated. T5293 validated via independet antibody verification, specific for human tubulin in IF; 715-005-151 and 715-005-152: tested with immunoelectrophoresis and ELISA for specificity and crossreactivity.

Eukaryotic cell lines

Policy information about cell lines

Cell line source(s)	U-2OS, homo sapiens bone osteosarcoma, ATCC-HTB-96, Lot # 61074667
Authentication	Cell lines were not further authenticated.
Mycoplasma contamination	cell lines were tested negative for mycoplasma
Commonly misidentified lines (See ICLAC register)	No commonly misidentified cell lines were used
